# Cancer antigen 125 assessment using carbon quantum dots for optical biosensing for the early diagnosis of ovarian cancer

**DOI:** 10.1039/d1ra05121k

**Published:** 2021-09-20

**Authors:** Walaa E. Omer, Mostafa F. Abdelbar, Nesma M. El-Kemary, Naoki Fukata, Maged A. El-Kemary

**Affiliations:** Institute of Nanoscience and Nanotechnology, Kafrelsheikh University 33516 Kafr Elsheikh Egypt elkemary@nano.kfs.edu.eg elkemary@yahoo.com; International Center for Materials Nanoarchitectonics, National Institute for Materials Science Tsukuba Ibaraki 305-0044 Japan; Department of Pharmaceutical Microbiology, Faculty of Pharmacy, Kafrelsheikh University 33516 Kafr Elsheikh Egypt; Graduate School of Pure and Applied Sciences, University of Tsukuba Tsukuba Ibaraki 305-8573 Japan

## Abstract

Fluorometric quantification of biological molecules is a key feature used in many biosensing studies. Fluorescence resonance energy transfer (FRET) using highly fluorescent quantum dots offers highly sensitive detection of the in-proximity wide variety of analyst molecules. In this contribution, we report the use of carbon quantum dots (CDs) for the ultrasensitive optical biosensing of cancer antigen 125 (CA-125) in the early malignant stage. This approach is based on monitoring the quenching of CDs luminescence at 535 nm by CA-125 after excitation at 425 nm and pH 10. The calibration of this method was performed in the concentration range of CA-125 from 0.01 to 129 U ml^−1^ (*R*^2^ = 0.99) with a detection limit of 0.66 U ml^−1^, which matches remarkably with the standard chemiluminometric method in control and real patient samples. The sensing mechanism for cancer antigen 125 assessment was discussed on the basis of fluorescence quenching of CDs and time-resolved photoluminescence spectroscopy. The current method is easy, sensitive, cost-effective and provides a wide range of validity, which helps in overcoming the limitations of high cost and time consumption exhibited by many other traditional clinical assays for CA-125 quantification.

## Abstract 2

Fluorometric quantification of biological molecules is a key feature used in many biosensing studies.

## Graphical abstract

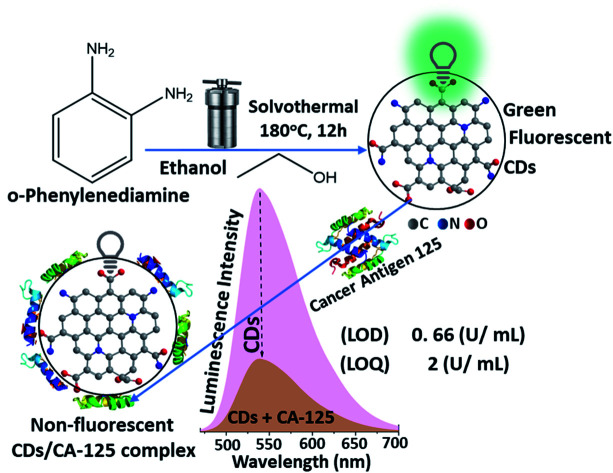

## 1. Introduction

Tumor antigen 125 (CA-125) is a surface antigen associated with non-mucinous epithelial ovarian cancer.^1^ The protein is produced on the surface of ovarian cancer cells and is released as soluble in serum or ascites.^2^ It belongs to the mucin family of glycoproteins, which includes about 22 000 amino acids and is encoded by the MUC16 gene in humans.^3,4^ It is elevated in the blood of patients with certain types of cancer or other benign diseases and therefore can be used as a tumor marker.^5^ The CA-125 test is one of the blood tests that can be ordered if ovarian cancer is suspected, as CA-125 levels can increase in benign conditions such as diverticulitis, endometriosis, cirrhosis of the liver, pregnancy, and uterine fibroids.^6^ In addition, this test is used to track a woman's response to treatment and to predict the patient's prognosis after treatment.^7^ Ovarian cancer remains the most common cause of death in women 20 years after diagnosis, as it often goes undetected until it has spread to the abdomen. In this advanced stage, ovarian cancer is more difficult to treat. Thus, early-stage ovarian cancer diagnosis, in which the disease is limited to the ovaries, is more likely to be treatable. This can be done by monitoring the concentration of CA-125.

Nanotechnology plays a significant role in the production of advanced sensor materials with high selectivity, sensitivity, and enhanced signal response.^8–10^ Fluorescent probes based on QDs have emerged in this contest due to their unique photoluminescence properties and accessibility for surface modifications.^11,12^ FRET between a QD sensor and analyzer molecules has been extensively studied in various biochemical research and biodetection platforms, including immunoassays, nucleic acid detection, clinical/diagnostic assays, and cell labeling.^13^ Recently, we have reported the use of a highly selective optical sensor for the assessment of total prostate-specific antigen in the serum of prostate cancer patients^14^ and cancer antigen CA 125.^15^

Carbon quantum dots (CDs) have attracted increasing scientific attention because of their promising optical properties, good photostability, low toxicity, excellent biocompatibility, easy synthesis, and the possibility of functionalization.^16,17^ Compared to graphene quantum dots that have low solubility in common solvents, the surface functional groups of CDs result in good solubility and chemical stability in aqueous and polar organic solvents.^18^ This new category of nanomaterials is being considered as a possible alternative to semiconductor QDs, which emit stable and bright luminescent colors in solution and in a polymer matrix under ultraviolet light excitation. Its ease of manufacture and unique optical properties make it potentially useful in biomedical fields such as optical bioimaging (*in vivo* and *in vitro*), cell labeling, drug delivery, gene delivery, cancer therapy and biosensors.^16^ These have also been used in various technologies, for example, in LEDs,^19^ photocatalysis,^20^ water splitting, and optoelectronic components.^21^

Although, there were a lot of traditional clinical assays for CA 125 quantification, such as enzyme-linked immune-sorbent and radiometric immunoassay that show high sensitivity and specificity but they are expensive, need several complicated operations and separation steps and, as such, are time-consuming.^22^ Thus, developing low-cost, chemically stable and photostable, as well as easily synthesized biocompatible nanosensor is an alternative challenge.

In this work, fluorescent CDs were synthesized from the orthophenylenediamine isomer by a simple solvothermal method and were used to quantify CA-125 in various serum samples from ovarian cancer patients. The evaluation process relies on the quenching of the luminescence intensity of the optical biosensor by various concentrations of CA-125 at 535 nm after excitation at 425 nm in ethanol at pH 10. Additionally, CDs have been embedded in a polymethyl methacrylate (PMMA) polymer matrix, resulting in a homogeneous, solid, and highly fluorescent biosensor thin film.

## 2. Experimental

### 2.1. Materials and reagents

The starting materials are *o*-phenylenediamine (flaked, 99.5%), polymethyl methacrylate (PMMA), HCl, NaOH, KCl, NaCl, uric acid, albumin, glucose, urea, triglyceride were purchased from Sigma-Aldrich. Tumor antigen 125 (CA-125, 130 U ml^−1^), CA 19-9 (carbohydrate antigen 19-9, 120 U ml^−1^), CA 15-3 (carcinoma antigen, 120 U ml^−1^) were purchased from (orb98857, Biorbyt). Working solutions for cancer antigen 125 and other biomarkers were prepared by liquefying the contents of a vial with a biomarker in 1 ml of deionized water and storing at 4.0 °C. Human samples were collected from the New Kasr El Aini Teaching Hospital at Cairo University and the Ain Shams Specialized Hospital of Ain Shams University, Cairo, Egypt, according to a protocol approved by WHO (World Health Organization) for collecting human samples and the use of these materials and associated clinical information for research purposes. All patients reported giving their consent and approved the use of their clinical specimens in the research work.

### 2.2. Apparatus

All equipments used in this study was made available at the Institute of Nanosciences and Nanotechnology, Kafrelsheich University, Egypt. The absorption spectra of the samples were measured in the range from 200 to 800 nm on a Shimadzu UV-2450 double-beam spectrophotometer. Luminescence measurements were performed on Shimadzu RF5301PC spectrofluorometer in the range (200–900 nm). FTIR spectra were recorded on a JASCO FTIR-6800 in the range from 400 to 4000 cm^−1^ using KBr pellets. The separation of proteins from samples was achieved by centrifuging the sample at 4000 rpm for 15 minutes. High-resolution imaging for morphological examination of the samples was performed using a JEOL JEM-2100 transmission electron microscope. The crystallinity and phase structure of the materials were investigated with an X-ray diffractometer (Shimadzu 6000-XRD) using Cu-Kα radiation (*λ* = 1.54056 Å). Zeta potential measurements were performed using Brookhaven Instruments. Measurements of picosecond emission decays were performed by a time-correlated single-photon counting technique (FluoTime 200, PicoQuant). The sample was excited by a 40 ps pulsed (20 MHz) laser centered at 375 nm (PicoQuant), and the emission signal was collected at the magic angle. The IRF of the apparatus was typically 65 ps. The apparatus and experimental details, as well as data analysis procedure, have been described elsewhere.^23^ All measurements were performed at 298 K.

### 2.3. General procedures

The synthesis of CDs optical sensors embedded in the PMMA matrix.

**Fig. 1:**
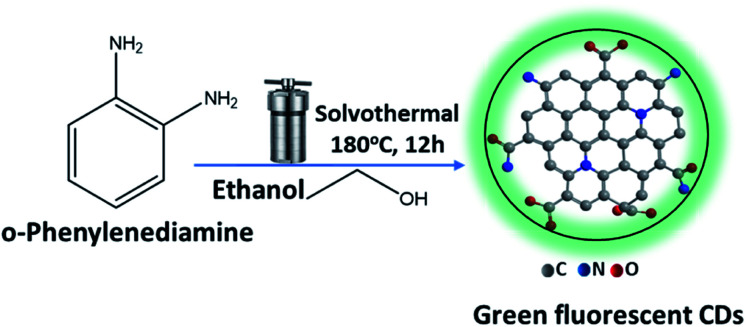
Reaction scheme for the synthesis of CDs with green fluorescence.

Luminescent CDs were prepared from (*o*-PD) *ortho* phenylenediamine by dissolving 1.0 g of *o*-PD in 90 ml of ethanol. The solution was then transferred to a Teflon autoclave and heated at 180 °C for 12 hours in an oven, Fig. 1. Then, the solution was cooled to room temperature and a dark orange suspension solution of CDs was obtained. The crude products were then purified by dialysis membrane (the dialysis bags molecular weight cutoff [MWCO] = 10 000 daltons) with an ethanol solution by placing on opposite sides of the membrane to obtain a clear yellow solution when dispersed in ethanol. After drying under vacuum to remove solvents, fine powders could be finally obtained.

Doping of the optical sensor CDs in PMMA polymer was done by adding 1.50 g PMMA to 10 ml CHCl_3_ with continuous stirring for 30 min until complete dissolution at 60 °C. Then, 200 μl of CDs (0.01 g ml^−1^) was added with continuous stirring for 30 minutes until a homogenous matrix was formed. The solution was finally cast in a 60 mm × 15 mm Petri-dish and kept at 25 °C until complete dryness. The thin film's width and height were 8.5 and 25 mm, respectively.

### 2.4. Sample preparation

Serum samples were taken from all volunteers; (i) control subjects (5 samples), (ii) ovarian cancer patients (7 samples). Then, 3 ml of citrate solution was added to 4.0 ml of plasma and the solution was centrifuged for 15.0 minutes at 4000 rpm to remove all proteins. After decantation, serum samples were obtained.

### 2.5. Proposed method

An appropriate amount (100 μl) of various standard samples of CA-125 in ethanol was mixed with a thin film of CDs in the PL cuvette. Luminescence spectra were recorded upon excitation at 425 nm. The optical biosensor was washed with ethanol after each measurement, and a calibration plot was made by plotting (*F*_0_/*F* − 1) at *λ*_em_ = 535 nm on the *y*-axis *versus* the concentration of CA-125 on the *x*-axis. The concentration of CA-125 was measured by taking (100 μl) of each serum sample and diluting to 2 ml with ethanol and 0.1 ml of phosphate universal buffer solution (pH 10) in the presence of an optical biosensor film in the spectrofluorimeter cuvette, then the emission intensity of the optical biosensor film was measured at 535 nm.

### 2.6. Standard method

The ADVIA Centaur CP CA-125 II assay is a two-site enzyme-linked immunosorbent assay using direct chemiluminometric technology based on using two mouse monoclonal antibodies specific for CA 125. The first antibody was directed against the M11 antigen domain and is labeled with acridinium ester. The second antibody targets the OC 125 antigen domain and was labeled with fluorescein. The immune complex formed with CA-125 was captured by the anti-fluorescein mouse monoclonal antibody, which binds to paramagnetic particles in the solid phase.

## 3. Results and discussion

### 3.1. Characterization of carbon quantum dots

**Fig. 2:**
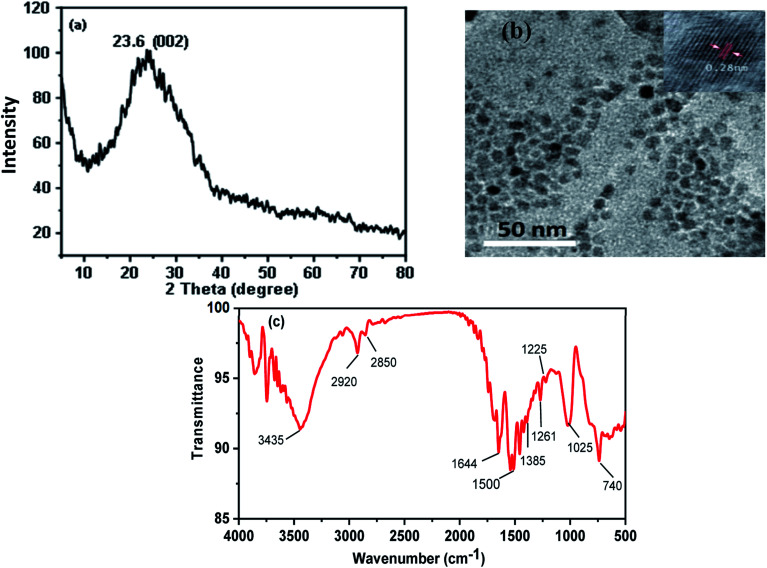
(a) XRD (b) TEM, and (c) FTIR spectrum of CDs.

The XRD pattern of CDs shows a single peak centered at 2*θ* = 23.6°, corresponding to the (002) plane, Fig. 2(a). The broad nature of the diffraction peak corresponds to the small size of CDs. Fig. 2(b) shows the TEM image of the as-synthesized CDs. It exhibited monodispersed quasi-spherical particle shapes and a size of about 6.0 ± 0.5 nm with an interlayer spacing (*d*-spacing) of 2.80 Å. The larger *d*-spacing value of the synthesized CDs suggests the presence of nitrogen functional groups.^24,25^ Fourier transform infrared (FT-IR) spectra were used to identify chemical bonds and surface functional groups on the CDs, Fig. 2(c). It indicates the presence of stretching vibration bands at 3435, 1644, 1225 and 1025 cm^−1^ corresponds to stretching bands of O–H, C=O of COOH, C–O, and C–O–C, respectively, and reveals the presence of carboxylic acid in CDs. Broad absorption peak in the range of 3250–3050 cm^−1^ and at 1261 cm^−1^ is associated with N–H and C–N of amino functional groups that existed in CDs.^26–30^ Additionally, three stretching absorption bands of C–H, C=C, and C–C are observed at 2920, 1450, and 1385 cm^−1^, respectively, suggesting the presence of alkyl and aryl groups.

**Fig. 3:**
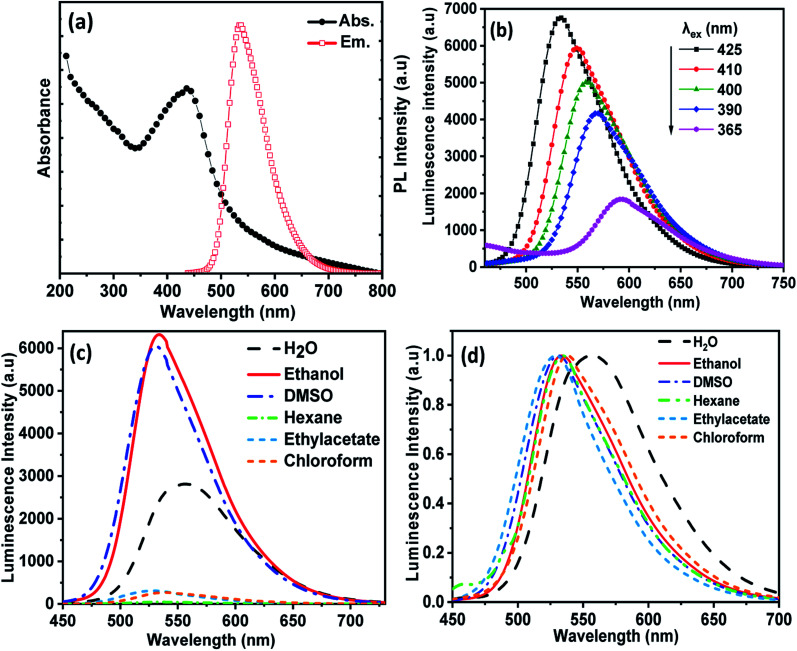
(a) Absorption and emission spectra of CDs, (b) the effect of different excitation wavelength on the emission spectra of CDs, (c and d) the effect of solvent on the emission spectra of CDs, where (d) shows the normalized emission intensity represented in (c).

The optical properties of CDs were monitored using UV-visible absorption and photoluminescence (PL) emission studies. As shown in Fig. 3(a), the UV-visible absorption spectra (left) of CDs in ethanol exhibited two absorption peaks. The observed absorption peak at ∼250 nm is corresponding to the π–π* transition of the aromatic π-system and the peak appeared at 425 nm originating from the n–π* transition of nitrogen-doped CDs. The PL spectra (right) of CDs in ethanol showed a maximum emission intensity at 535 nm upon excitation at 425 nm. Fig. 3(b) shows the effect of excitation wavelengths on the PL emission of CDs in ethanol, which exhibits excitation wavelength dependence. As the excitation is decreased from 425 nm to 365 nm, the position of the strongest emission peak shifts from 535 nm to 595 nm with a gradual decrease in intensity. Thus, CDs exhibited multicolors such as green, blue, yellow and red when excited with different excitation wavelengths. This feature agrees well with other reports and it was attributed to the optical selection of various states of emission traps close to the CDs Fermi level and different sizes of nanoparticles (quantum effect).^31,32^

The effect of different solvents on the PL spectra of CDs at a single excitation wavelength (*λ*_max_ = 425 nm) is shown in Fig. 3(c) and (d). The peak of CDs emission in water was observed at 555 nm, and in ethanol at 535 nm. Therefore, the emission peaks show a red shift with increasing solvent polarity. The phenomenon of solvent-dependent PL in CDs is based on solvatochromism, which is widely used in organic dyes and is usually associated with intramolecular charge transfer.^33,34^ As the polarity of the solvent increases in aprotic solvents, the dipole moment increases, and the effect of surface composition on the electronic structure increases. Secondly, it lowers the energy of the CD band gap and causes a red shift of the fluorescence emission wavelength.^35^ The maximum CDs luminescence intensity was observed in ethanol, Fig. 3(c), since functional groups on the CDs surface, such as nitrogen and oxygen atoms, can form strong hydrogen bonds with alcohol molecules, which leads to the stabilization of the excited state of CDs, although water molecules inhibited the PL intensity of CDs due to poor water solubility and subsequent aggregation of CDs.^36^

**Fig. 4:**
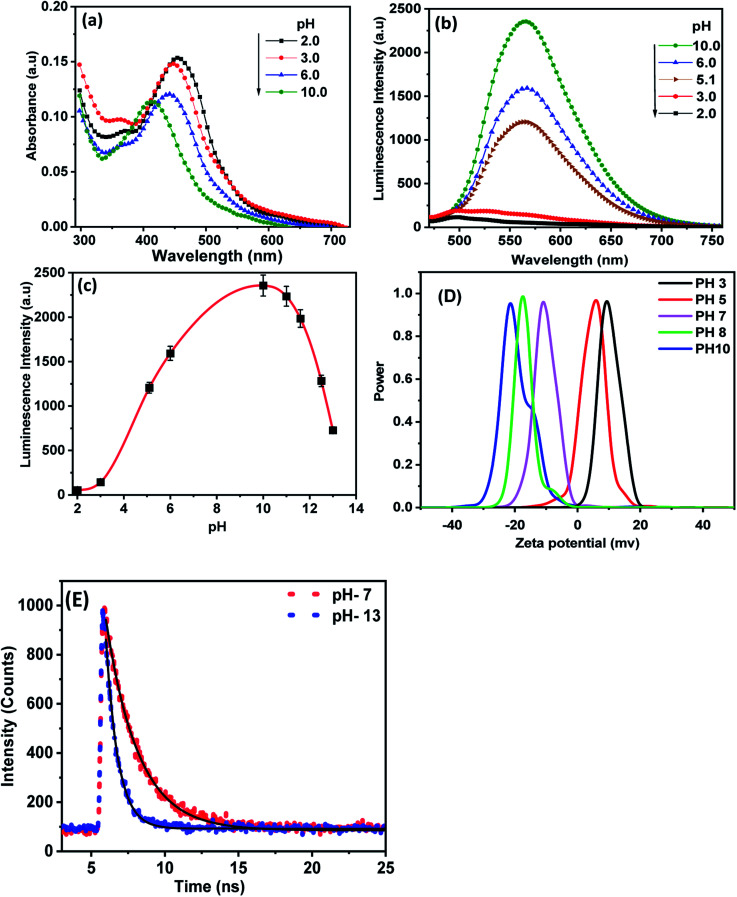
The effect of pH on the (a) absorption and (b) emission spectra of CDs, (c) the effect of pH (2–13) on the intensity of emission of CDs, (d) zeta potential of the synthesized CDs at different pH and (E) the excited state lifetime decay of CDs at pH 7 and 13.

Fig. 4(a) shows the effect of the environmental pH on UV-visible spectra of CDs. It is readily seen that the absorption spectra of CDs exhibited a redshift from 413 nm to 455 nm with decreasing pH from 10 to 2.0. The obtained results suggest that in the acidic medium the amino acid and carboxyl groups on the surface are protonated and attract electrons of the π-system conjugated to CDs through a covalent bond. This behavior reduces the band gap of the CDs, and the absorption wavelength shifts towards a longer wavelength.^37^ As the pH increases, the surface functional groups of CDs are not protonated, leading to the enhancement of the electron density of CDs by a lone pair of electrons conjugated to the π-system, and then increases the bandgap energy resulting in a blue shift of the absorption wavelength.^38^Fig. 4(b) and (c) shows the changes in PL spectra of CDs at different pH values upon excitation at 425 nm. A considerable enhancement in the PL intensity of CDs was observed upon increasing the pH from 2.0 to 10. At pH above 10, the PL intensity decreases again. These spectral changes are probably due to the deprotonation of the functional groups in a highly alkaline medium, which causes the aggregation of CDs, and therefore fluorescence quenching.^39^ In view of the above results, it is possible to say that highly positively or negatively charged surface groups on highly acidic and basic mediums tend to quench luminescence compared to neutral groups.

To gain insight into the pH-dependent surface charge of the as-synthesized CDs, we measured the zeta potential of CDs at different pH values. As shown in Fig. 4(d), the zeta potential values of the as-synthesized CDs exhibited positive values of 9.5 and 5.8 in acidic pH of 3 and 5, respectively. However, a negative charge of −11, −17.5 and −21.5 was observed at pH values of 7, 8 and 10, respectively. This suggests better stability of the current CDs in an alkaline medium. This result demonstrates that the stability of CDs increases with increasing pH value, which is in close agreement with the photoluminescence characteristics of CDs at various pH values.

Fig. 4(e) shows the time-resolved fluorescence spectra of CDs in aqueous media of pH values 7 and 13 upon excitation at 425 nm. The decay recorded at 530 nm was fitted to one exponential function with lifetimes 1.9 ns and 0.66 ns at pH values 7.0 and 13, respectively. The decrease in lifetime with increasing pH value can be explained as a result of the absorption of OH in the basic medium, which creates a protective coating on the initial acid functional groups on the CD's surface. Therefore, the CDs become non-isolated and the rate of nonradiative recombination increases.^40,41^

### 3.2. Analytical parameters

**Fig. 5:**
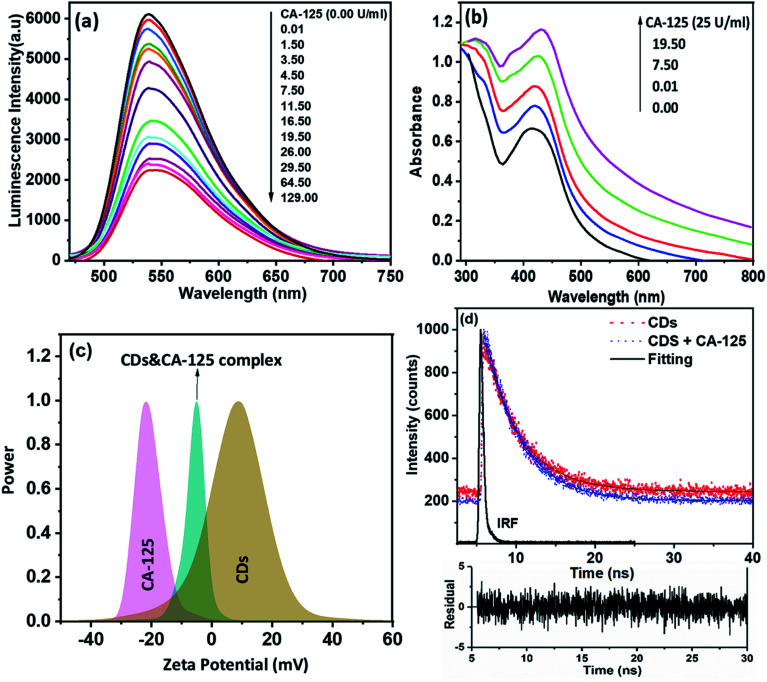
(a) Fluorescence emission spectra of CDS in different concentration of CA-125 protein (*λ*_ex_ = 425 nm), (b) the effect of gradual addition of CA-125 protein on the absorption spectra of CDs, (c) zeta potential of CDs, CA-125 protein, and CDs & CA-125 complex, and (d) excited state lifetime decay of CDs and CDs with CA-125.

The effect of CA-125 concentration on the fluorescence intensity of CDs' optical biosensing was investigated in ethanol at pH 10, as shown in Fig. 5(a). The detection process was based on the quenching of the fluorescence intensity of the CD's optical biosensing by increasing the concentration of CA-125 to 129 U ml^−1^. Fig. 5(b) shows that the UV-vis absorption spectra of CA-125 have absorption bands at about 310 and 360 nm. The synthesized CDs exhibited an emission band of approximately 535 nm when excited at 425 nm. There is a small overlap between the absorption spectrum of CA-125 and the emission spectra of the CDs as indicated in Fig. 7(d). Consequently, the fluorescence quenching of CDs can occur by a static mechanism with a small contribution to FRET.^42,43^ The absorption spectra of CDs before and after the addition of CA-125 give a clear idea of the binding mechanism between CDs and CA-125, while the formation of a complex in the ground state can lead to a change in the absorption spectrum of CDs.^44^ As shown in Fig. 5(b), there was a weak peak appearing around 320 nm in addition to a small red-shift in the absorption maxima (∼12 nm) accompanied by an increased absorption intensity with increasing CA-125 concentration. The spectral changes can be attributed to the formation of the ground state complex, which was assisted by the opposite charge accumulated on surfaces of CDs and CA-125 as shown from the zeta potential measurements Fig. 5(c). Consequently, the binding of CDs with CA-125 was accompanied by a formation of the non-fluorescent complex, which explains the sequential quenching of CDs by CA-125.

Based on the small overlap between the absorption of CA-125 and the emission of CDs, the FRET contribution still exists even though it cannot completely deactivate the fluorescence of CDs and remarkably affect the excited state dynamics of CDs. This type of energy transfer is mainly a static type, while, in static quenching, the fluorescence lifetime does not change (*τ*_0_/*τ* = 1), where *τ*_0_ and *τ*, are the fluorescence lifetimes of CDs in the absence and presence of CA-125, respectively. Fig. 5(c) shows the zeta potential values for CDs, CA-125, and complex between CA-125 and CDs to be 9 mV, −21 mV, and −5 mV, respectively. The relatively higher negative charge of CA-125 can be attributed to the deprotonated COO^−^ groups of the protein at pH slightly higher than the isoelectric point of the protein (IEP = 6.2–7.3). In the case of CDs dissolved in ethanol, a broad zeta potential peak centered at +9 mV was observed. Taking into account the nanostructured nature of CDs, where both surface and bulk amino, and carboxylic groups have broad and different IEPs from protein. Therefore, there was an electrostatic attraction between the oppositely charged CDs and CA-125. The net charge on the formed complex is dependent on the concentration of added CA-125. Fig. 5(d), illustrates the fluorescence lifetime of CDs in the absence and presence of different concentrations of CA-125 in the buffer solution of pH 10 was nearly the same (4.32 and 4.30 ns). Therefore, *τ*_0_/*τ* = ∼1. So, the mechanism of fluorescence quenching is static.^45^

**Fig. 6:**
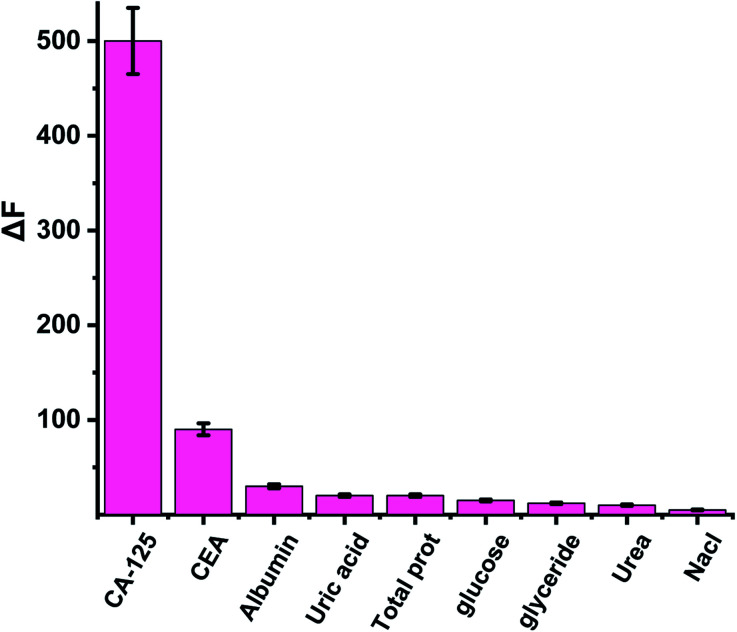
The effect of interfering species on the luminescence spectrum of CDs.

The selectivity and the validity of the proposed method were checked by studying the influence of a number of interfering species on the fluorescence spectrum of CDs. For example, CA 19-9 (130 U ml^−1^), CEA (130 U ml^−1^), CA 15^−3^ (130 U ml^−1^), KCl, NaCl (2.0 × 10^−3^ U ml^−1^), albumin (0.7 g l^−1^), urea (0.06 g l^−1^), uric acid (0.08 g l^−1^), total protein (0.01 g l^−1^), glucose (0.08 g l^−1^) and (0.06 g l^−1^) triglycerides. The tolerance limit was defined as the concentration of individually added substances that causes a deviation of less than 3% of the fluorescence intensity under optimal conditions of a thin film of the optical CD sensor. Δ*F* was calculated to compare the interaction of CA 125 and various interfering particles with an optical biosensor (Δ*F* is the luminescence intensity of the optical sensor in the absence of interfering particles – the luminescence intensity of the optical sensor in the presence of interfering species). The results showed that interfering species had no significant effect on the fluorescence intensity of CDs with CA-125 (Fig. 6).

## 4. Method validation

### 4.1. Dynamic range

**Fig. 7:**
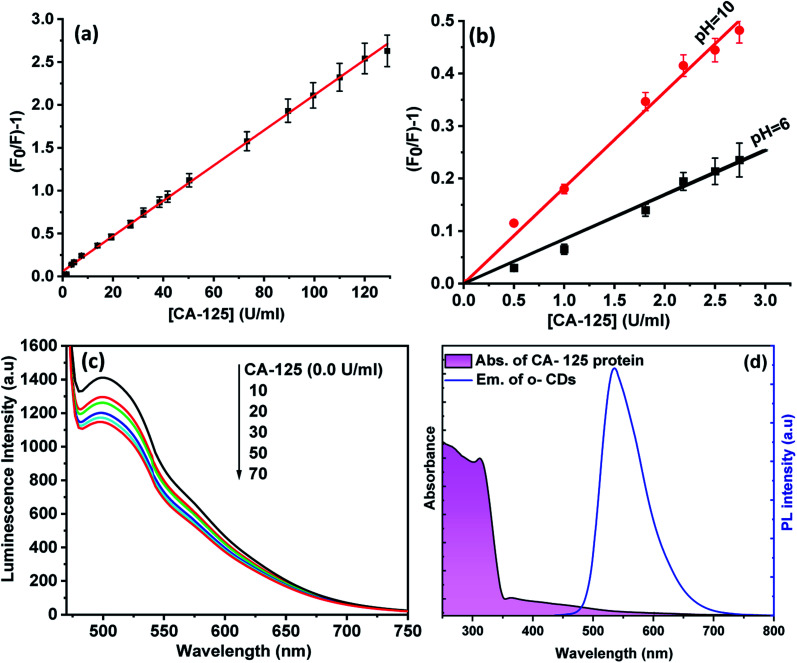
(a) The Stern–Volmer plot for the CDs quenching by CA-125 (b) the effect of pH on the quenching of CDs by CA-125 illustrated by the Stern–Volmer plot at pH 6, and 10, (c) the quenching of CDs embedded in PMMA film by CA-125 and (d) spectral overlap between absorbance of CA-125 and emission of CDs.

The Stern–Völmer equation was applied to study the influence of CA-125 concentration on the fluorescence intensity of CD optical sensor:^46^ (*F*_0_/*F*) − 1 = *K*_SV_[Q], where *F*_0_ and *F* are the fluorescence intensities of the optical sensor in the absence and in the presence of CA-125, respectively, [Q] is the concentration of CA-125, and *K*_SV_ is the Stern–Völmer (SV) constant. As shown in Fig. 7(a), the SV plot is linear and the slope of the fitted data is equal to *K*_SV_ = 0.050 U ml^−1^. 1/*K*_SV_ is equal to *C*_1/2_ (half quenching concentration), *C*_1/2_ = 1/*K*sv = 20 U ml^−1^. The critical transfer distance (*d*_0_), is the distance at which the probability of intermolecular energy transfer is exactly equal to the sum of the probabilities for all processes of de-excitation of the excited state of the donor. *d*_0_ = 7.35/(*C*_1/2_)^1/3^ = 2.7 Å, [*R*_0_ < 10 Å] means that the quenching of CDs occurs *via* a static mechanism. The plot of [(*F*_0_/*F*) − 1] *versus* [CA-125] shows that the fluorescence intensity at 535 nm decreases linearly with [CA-125] in the concentration range from 0.01 to 128 U ml^−1^. The correlation coefficient is 0.989. The detection limit (LOD) and quantification detection limits were calculated according to ICH guidelines^47^ using the formulas: LOD = 3.3*Sb* and LOQ = 10*Sb* (where *S* is the standard deviation of the blank fluorescence intensity values, and *b* is the slope of the calibration graph). The results are summarised in Table 1.

**Table 1:** Sensitivity and regression parameters for the proposed method

Parameter	Values
*λ*_em_ (nm)	535
Linear range (U ml^−1^)	0.01–128
Limit of detection (LOD) (U ml^−1^)	0.66
Limit of quantification (LOQ) (U ml^−1^)	2.0
Intercept (*a*)	0
Slope (*b*)	0.050
Standard deviation	0.01
Regression coefficient (*R*^2^)	0.989

The comparison of the results of dynamic range and LOD obtained by the proposed method with those from the reported methods for the determination of CA-125, Table 2, indicates that the developed method has good stability, a lower detection limit (0.66 U ml^−1^) and a wide linear range of application (0.01–128 U ml^−1^).

**Table 2:** Comparison of the proposed method sensitivity with other sensors for the determination of CA-125

Method	Linear range (U ml^−1^)	Detection limit (U ml^−1^)	Ref.
Up-conversion fluorescence biosensor	5–100	120	48
Electrochemiluminescence based optical biosensing	0.001–5	0.64	49
Surface plasmon resonance based optical biosensing	1.0–80	0.64	50
Colorimetric based optical biosensing	0.1–100	0.71	51
Carbon quantum dots as optical biosensor	0.01–128	0.60	This work

Fig. 7(b) shows the SV plots for quenching of CDs by CA-125 at pH 10 and 6. At pH 10, the slope of the linear fit is larger than that at pH 6, and quenching of luminescence is more sensitive for detection of CA-125 at pH 10. This observation can be explained by taking into account that at pH 10, the surface of CA-125 becomes more negatively charged due to the deprotonation of the functional group, therefore the electrostatic interaction can be enhanced. The CDs were encapsulated in a PMMA matrix to produce a homogeneous, transparent, and fluorescent solid film. This film shows the PL emission band at 502 nm upon excitation at 425 nm. Fig. 7(c). A blue shift in the PL peak of the CD film was observed as polymers around the CDs particles exerted similar interactions with the surface of the CDs and therefore change their surface electronic structures and bandgap of CDs. Different concentrations of CA-125 protein were added to the film in ethanol solution in fluorescence cuvette. The PL of CDs film was quenched by increasing CA-125 concentration and the observed quenching parameters were almost the same as in colloidal CDs. This may be attributed to the higher chance of protein aggregation on the surface of the film, which can enhance FRET efficiency.

### 4.2. Accuracy and precision of the method

To assess accuracy and precision, the tests described in the general procedures were repeated three times daily to determine repeatability (intraday precision) and three times on different days to determine the intermediate precision (inter-day precision) of the method. These analyses were performed on twelve samples. The results of this study are presented in Table 3. The percentages of relative standard deviation (RSD%) were ≤0.005–0.065 (serum) (within one day), ≤0.004–0.095% (serum) (inter-day), indicating a high precision of the method. Accuracy was assessed as the percentage of relative error (RE) between the measured mean concentrations and the taken CA-125 concentrations. Bias {bias% = [(found concentration − known concentration) × 100/known concentration] was calculated for each concentration, and these results are also shown in Table 3. The values of the relative percentage error (RE%) ≤−3.33 to 0.43% (serum) (intra-day) and ≤−5 to 3.91% (serum) (inter-day during the day) show high accuracy of the proposed method.

**Table 3:** Evaluation of intra-day and inter-day accuracy and precision of the proposed method in case of different serum samples^a^

Serum samples	Standard method	Proposed method							
		Intra-day accuracy and precision (*n* = 3)				Inter-day accuracy and precision (*n* = 3)			
	Average	Average reading	±CL	RE (%)	RSD (%)	Average reading	±CL	RE (%)	RSD (%)
Patient (1)	10	10.2	±0.0135	2.00	0.98	10.5	±0.0133	5.00	0.95
Patient (2)	23	22.9	±0.0090	0.43	0.44	22.1	±0.0092	3.91	0.45
Patient (3)	27	27.1	±0.0083	0.37	0.37	28	±0.0081	3.70	0.36
Patient (4)	15	15.5	±0.0109	3.33	0.65	14.5	±0.0113	3.33	0.69
Patient (5)	18	18.3	±0.0101	1.67	0.55	18	±0.0101	0.00	0.56
Patient (6)	99	98.8	±0.0043	0.20	1.00	100	±0.0043	1.01	1.00
Patient (7)	78	78.0	±0.0049	0.00	1.30	79	±0.0048	1.28	1.31
Patient (8)	130	130.6	±0.0038	0.46	0.80	129	±0.0038	0.77	0.80
Patient (9)	210	210.2	±0.0030	0.10	0.50	212	±0.0030	0.95	0.50
Patient (10)	105	105.4	±0.0042	0.38	0.90	103	±0.0042	1.90	1.00
Patient (11)	222	221.7	±0.0029	0.14	0.50	225	±0.0043	1.96	0.10
Patient (12)	102	102.3	±0.0043	0.29	0.10	100	±0.0029	1.35	0.40

a RE: percent relative error, RSD (%): relative standard deviation and CL: confidence limits were calculated from: CL = ±*tS*/√*n* (the tabulated value of *t* is 4.303, at the 95% confidence level; *S* = standard deviation and *n* = number of measurements).

## 5. Conclusions

In this work, we have developed a fluorescence quenching method for the determination of CA-125, which offers an excellent approach to create high-quality biomarkers for early detection of ovarian cancer. The method relies on measuring the fluorescence intensity of both colloidal CDs and polymer embedded, which exhibits a remarkable quenching upon interaction with CA-125 under optimized conditions. The quenching mechanism was found to be driven by a ground-state complex, facilitated by the electrostatic attraction of CA-125 and CDs in addition to Förster resonance energy transfer FRET. Due to the lower detection limit of 0.66 U ml^−1^, the method is more sensitive than the standard approach.

## Conflicts of interest

There are no conflicts to declare.

## Supplementary Material
